# Estrogen promotes tumor metastasis via estrogen receptor beta-mediated regulation of matrix-metalloproteinase-2 in non-small cell lung cancer

**DOI:** 10.18632/oncotarget.16992

**Published:** 2017-04-10

**Authors:** Sheng Fan, Yongde Liao, Changyu Liu, Quanfu Huang, Huifang Liang, Bo Ai, Shegnling Fu, Sheng Zhou

**Affiliations:** ^1^ Department of Thoracic Surgery, Tongji Hospital, Tongji Medical College, Huazhong University of Science and Technology, Wuhan 430030, Hubei Province, China; ^2^ Hepatic Surgery Center, Tongji Hospital, Tongji Medical College, Huazhong University of Science and Technology, Wuhan 430030, Hubei Province, China; ^3^ Department of Pathology, Tongji Hospital, Tongji Medical College, Huazhong University of Science and Technology, Wuhan 430030, Hubei Province, China

**Keywords:** non-small cell lung cancer (NSCLC), estrogen, estrogen receptor beta (ERβ), matrix-metalloproteinase (MMP), metastasis

## Abstract

In non–small cell lung cancer (NSCLC), estrogen significantly promotes NSCLC cell growth via estrogen receptor beta (ERβ). However, the effects by which ERβ contributes to metastasis in NSCLC have not been previously reported. This study aims at defining whether the stimulation of ERβ promotes NSCLC metastasis *in vitro* and *in vivo*. Here, Our results showed that estrogen and ERβ agonist enhanced aggressiveness of two lung cancer cell lines (A549 and H1793) and promoted murine lung metastasis formation. ER-inhibitor Fulvestrant treatment or ERβ-knockdown significantly suppressed the migration, invasion and nodule formation of NSCLC cells. The expression level of ERβ protein was analyzed in matched samples of metastatic lymph node and primary tumor tissues from the same individuals, and we found significantly higher levels of ERβ were expressed in lymph node compared to primary tumor tissues. Moreover, Studies on both surgical biopsies and on lung cancer cells revealed that the expression level of ERβ and matrix-metalloproteinase-2 (MMP-2) were associated. Furthermore, inhibition of ERβ resulted in down-regulation of MMP-2 expression. Taken together, our results demonstrate that activation of ERβ in lung cancer cells promotes tumor metastasis through increasing expression of invasiveness-associated MMP-2. These results also highlight the therapeutic potential of inhibition of ERβin the treatment of advanced NSCLC.

## INTRODUCTION

Non-small cell lung cancer (NSCLC) is one of the most prevalent malignant tumors and is the leading cause of cancer-related death worldwide [1]. Despite early diagnosis, mortality rates in NSCLC patients remain overly high, with the 5-year overall survival rate of only 15%. During the course of disease, metastasis occurs in approximately 40% to 50% of patients with advanced NSCLC, and the survival time is less than 6 months [2, 3]. According to recent epidemiological data, metastatic NSCLC contributes to rising morbidity and increasing mortality. Indeed, over 70% of advanced NSCLC patients die from metastatic spread [4]. However, there is no effective therapeutic target in patients with advanced metastatic lung cancer. Therefore, identification of potential therapeutic targets is a priority to prolong the survival of lung cancer patients.

Estrogen has been reported to be an important driver of NSCLC in recent years [5]. A study of over 16,000 postmenopausal female patients in the Women's Health Initiative found that women who received daily hormone replacement therapy (HRT) over 5 years showed obviously higher lung cancer incidence and mortality than the placebo group [6]. However, anti-estrogen treatment significantly decreased the risk of lung cancer mortality in HRT patients, in another cohort study consisting of 6655 women diagnosed with breast cancer [7].

The biological effects of estrogen are mediated via binding to structurally and functionally distinct estrogen receptors, alpha and beta (ERα and ERβ) [8]. Several studies have established a positive role of ERα signaling in the motility of metastatic tumors retaining ERα [9]. More than 80% of lymph node metastases and 65% to 70% of distant metastases overexpressed ERα in the breast cancer [10, 11]. In contrast to breast cancer, ERβ stimulated by estrogen is a major ER subtype in NSCLC [5, 12]. Recent reports suggest that estrogen induces NSCLC cell proliferation *in vitro* [13] and affects human tumor xenografts [12]. Additionally, our previous study also demonstrated that estrogen significantly upregulated IGF-1R expression via ERβ and promoted murine lung adenocarcinoma [14, 15]. ERβ acts as a transcription factor by binding with 17β-estradiol (E2). Upon nuclear translocation, it binds to estrogen responsive element (ERE) within the target gene promoters, and stimulates gene transcription [16, 17]. In addition to the nuclear signaling pathway, extranuclear signaling has been linked to rapid responses via E2-mediated stimulation of the Src kinase, mitogen-activated protein kinase (MAPK), protein kinase B (AKT), phosphatidylinositol-3-kinase (PI3K), PKA and PKC pathways in the cytosol [18, 19]. In brief, our previous study has confirmed the role of ERβ in cell proliferation and crosstalk with grow factors. However, the role of ERβ in NSCLC progression has not been fully investigated.

Metastases triggered by epithelial cancers are multi-stage processes involving invasion into surrounding tissue, intravasation, transit in the blood or lymph, extravasation, and growth at a new site [20]. Frequently, matrix-metalloproteinases (MMPs) play an important role in local tumor invasion via the basement membrane and stroma [21]. Evidence suggests that NSCLC tumors express increased levels of MMP-1, MMP-2 and MMP-9 [22]. Single nucleotide polymorphisms (SNP) in MMP-9 are significant predictors for lung cancer development and MMP-2 polymorphisms predict overall survival [23]. However, the effects of ERβ in the progression of NSCLC metastasis and its relationship with MMPs are unknown.

In the present study, we focused on the effects of ERβ induced by estrogen in promoting metastasis of NSCLC and its possible mechanism. We first analysed the protein levels of ERβ, MMP-1, MMP-2 and MMP-9 and their connections. Immunohistochemistry and western blot by matched metastatic lymph node and primary tumor tissure was performed. We also demonstrate the metastatic malignant properties of two NSCLC cells which was treated with estradiol (E2), the ERα-selective agonist PPT, the ERβ-selective agonist DPN and the ER antagonist fulvestrant, ERβ-Knockdown or ERβ-Overexpressed. Furthermore, we developed a novel mouse model of NSCLC lung metastasis to investigate whether estrogen induced metastasis of A549 cells and protein expression *in vivo*. Finally, we verified the signal transduction pathway underlying this process.

## RESULTS

### Increased ERβ expression in NSCLC correlates with MMP-2 expression

**Figure 1:**
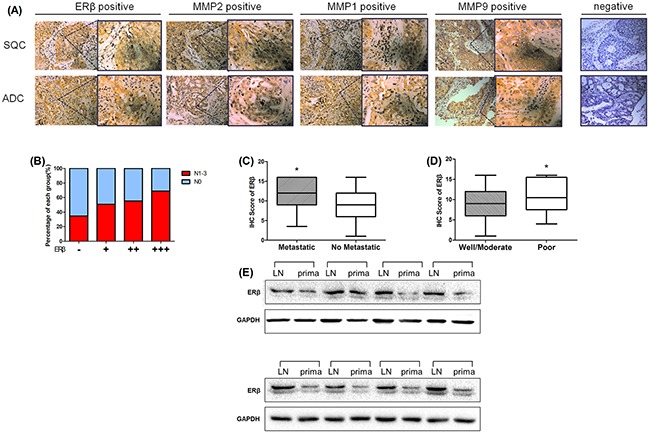
The expression of ERβ, MMP-1, MMP-2 and MMP-9 was elevated in paraffin-embedded NSCLC sections **(A)** Immunohistochemical analyses of ERβ, MMP-1, MMP-2 and MMP-9 in NSCLC tissue. NSCLC specimens were immunostained with ERβ, MMP-1, MMP-2 and MMP-9-specific antibodies, as mentioned in ‘‘Materials and methods’’ section. During immunostaining, positive cells appeared as yellowish brown or contained yellowish brown granules (Original magnification 200×(left) and 400×(right)). **(B)** The proportion of lymph node metastasis in NSCLC patients with different ERβ IHC scores as described in the Materials and Methods section (-: > 4; + : 4∼8; ++ : 8∼12; +++ : 12∼16); **(C)** Immunohistochemical staining scores in 222 NSCLC samples expressed by box plots. Significance of mean differences in staining scores between “Metastasis” and “No Metastasis” group is shown (*p = 0.017 < 0.05). **(D)** Significance of mean differences in staining scores between “Well/Moderate” and “Poor” tumor differentation grade group (*p = 0.035 < 0.05). **(E)** Western blot analysis of ERβ expression in 8-pair cases of metastatic lymph node tissues and primary NSCLC tumor tissues. GAPDH was used as a control.

The expression of ERβ in NSCLC tissues and corresponding normal lung tissues of 60 patients were reported in our previous study [14, 24]. In this study, we expanded the NSCLC sample to 222 patients in TMA and evaluated the expression of ERβ using immunohistochemistry. The results indicate that human estrogen receptor was negative in pneumocytes of normal lung tissues but positive in tumor tissues (Figure 1A). Positive ERβ expression accounted for 84.68% of all the specimens in patients with NSCLC (188 of 222 patients). The correlation between ERβ expression and clinicopathological features is shown in Table 1. The high expression of ERβ significantly correlated with clinically distant metastasis in NSCLC patients (p = 0.017) and poorly differentiated NSCLC patients (p = 0.035). However, other clinicopathological features including gender, age, smoking status, tumor stage, lymph node metastasis, tumor histology and pTNM stage were not directly associated with the expression of ERβ. However, interestingly, when we grouped the samples according to the distinct IHC score, we found that the proportion of patients with lymph node-metastasis increased with the ERβ IHC score (Figure 1B). Additionally, the different metastatic states and tumor grades are shown in Figure 1C-D.

**Table 1:** Correlation of ERβ overexpression with clinicopathological features in 222 cases of non-small cell lung carcinoma

	No. of patients(%)	ERβ Expression		Median IHC score of ERβ	χ^2^	*p* value
		positive	negative			
All	222					
Gender						
Female	80(36.03)	65	15	8.13	1.138	0.333
Male	142(63.96)	123	19	9.46		
Age						
<60	135(60.81)	112	23	8.75	0.787	0.375
≥60	87(39.18)	76	11	9.68		
Smoking						
Ex	139(62.61)	121	18	9.36	1.604	0.205
Never	83(37.38)	67	16	8.70		
T stage						
1a-2b	164(73.87)	138	26	9.10	0.140	0.708
3-4	58(26.13)	50	8	9.15		
Lymph node metastasis						
YES	146(65.77)	126	20	9.38	1.750	0.186
NO	76(34.23)	62	14	8.77		
Metastasis						
YES	17(0.54)	11	6	9.25	5.666	0.017
NO	205(94.60)	177	28	7.47		
TNM stage						
I-II	116(52.25)	97	19	8.75	0.212	0.645
III-IV	106(47.75)	91	15	9.51		
Tumor Histology						
SQC	108(48.65)	92	16	8.93	0.041	0.840
ADC	114(51.35)	96	18	9.30		
Tumor differentation						
Well/Moderate	88(39.63)	69	19	7.80	4.427	0.035
Poor	134(60.37)	119	15	9.98		

MMP-1, MMP-2 and MMP-9 are associated with local lymph node invasion or distant metastasis. Therefore, we also examined MMPs expression in NSCLC TMA mentioned above. We found that the percentage of ERβ-positive stained cells was significantly correlated with that of MMP-2-positive stained cells (Spearman's rho correlation coefficient = 0.428, p < 0.001; Table 2). However no significant correlation was observed between MMP-1 or MMP-9 and ERβ IHC score (MMP-1, correlation coefficient = - 0.125, p = 0.062; MMP-9, correlation coefficient = 0.033, p = 0.628; Table 2). These data suggest that the elevated expression of ERβ predicts tumor metastasis or poor tumor differentiation grade and high expression of ERβ was correlated with MMP-2 in human NSCLC.

**Table 2:** Correlation between ERβ expression with MMP-1, MMP-2 and MMP-9 in 222 cases of non-small cell lung carcinoma

MMPs	Expression	ERβ			
		Positive	Negative	P-value	Sperman's rho
MMP-2	Positive	115	11	<0.001	0.428
	Negative	73	23		
MMP-1	Positive	92	17	0.062	−0.125
	Negative	96	17		
MMP-9	Positive	85	18	0.628	0.033
	Negative	103	16		

### ERβ expression was significantly higher in metastatic lymph node than in primary NSCLC tumor tissue

**Figure 2:**
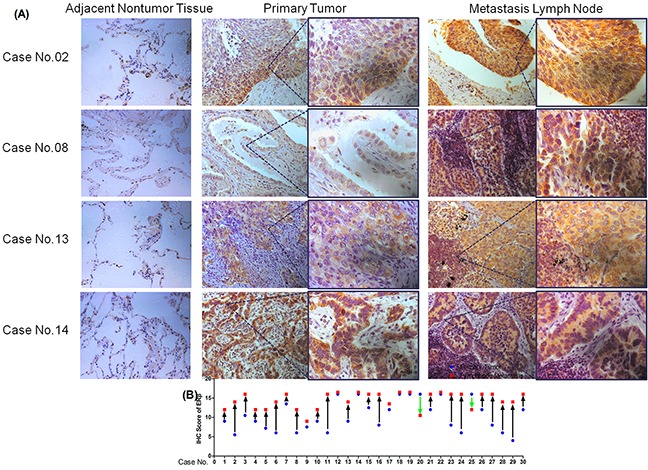
Expression of ERβ via immunohistochemical analyses of adjacent nontumor tissue **(A)** Primary NSCLC tumor tissues and metastatic lymph node tissues in four typical cases (Case No.02, No.08, No.13, No.14; Original magnification 200× (left) and 400× (right)); **(B)** IHC scores of the 30 pairs of specimens from both primary tumor tissue and metastatic lymph nodes (□: lymph nodes metastasis; △: primary tumor).

To elucidate the relationship between ERβ expression in primary tumors and lymph node metastases from the same NSCLC patient, we evaluated 60 samples in the paired primary tumors and lymph node metastases using immunohistochemical analysis. A total of 30 patients with lymph node metastasis (N1-N3) is shown in Table 3. Although positive ERβ expression was found in these 60 samples, the extent of positive expression varied in metastatic lymph node and primary tumor. Primary tumors in 7(23.33%) NSCLC patients expressed weakly positive ERβ, 14(46.67%) primary tumor specimens were moderately positive for ERβ while the other 9(30%) specimens were strongly positive. Eight (26.67%) NSCLC metastatic lymph node tissues expressed moderately positive ERβ and the other 22(73.33%) metastasis lymph nodes were all strongly positive. None of the metastatic lymph nodes expressed ERβ weakly. The Paired Samples t-test clearly showed that ERβ expression in metastatic lymph nodes was significantly higher than in primary tumor tissue (t = 4.402, p < 0.001, Table 3). Each patient's ERβ IHC scores of primary tumor and metastatic lymph node are shown in Figure 2. However, the expression of MMP-1, MMP-2, and MMP-9 was not different between primary NSCLC tumor and metastatic lymph node (Table 3, MMP-1 and MMP-9 not shown).

**Table 3:** List of biopsies included in this study with patient clinicopathologic parameters and immunohistochemical staining scores for ERβ and MMP-2

Case No.	Gender	Age at time of surgery	Tumor Stage UICC	Tumor Stage TNM	Lymphnode metastasis ratio	IHC score of ERβ		IHC score of MMP2	
						Primary Tumor	Lymphnode Metastasis	Primary Tumor	Lymphnode Metastasis
01	Female	48	IIIA	pT2aN2	11/13	++	++	+	+
02	Male	64	IIIA	pT2aN2	11/26	+	+++	+	-
03	Male	66	IIIA	pT2bN2	5/21	++	+++	-	-
04	Male	21	IIIA	pT2bN2	6/26	++	++	-	-
05	Male	63	IIIA	pT2bN2	4/16	++	++	++	+
06	Male	57	IIIA	pT2aN2	7/16	+	+++	+	-
07	Male	46	IIIA	pT2aN2	5/27	+++	+++	-	++
08	Female	58	IIIB	pT3N3	4/14	+	+++	+++	+
09	Male	52	IIIA	pT2aN2	5/18	++	++	-	-
10	Male	38	IIIB	pT4N2	4/16	++	++	-	-
11	Male	60	IIB	pT2bN1	2/40	+	+++	+	-
12	Male	58	IIIA	pT3N2	11/18	+++	+++	-	++
13	Male	53	IIIB	pT2bN3	14/20	++	+++	-	++
14	Female	57	IIIB	pT1bN3	9/12	+++	+++	+	+
15	Male	42	IIIA	pT2aN2	14/32	+++	+++	+	+
16	Male	72	IIIA	pT2aN2	7/11	++	+++	+	+
17	Male	64	IIB	pT2aN1	1/10	++	++	+	++
18	Male	61	IIIA	pT2bN2	2/10	+++	+++	++	++
19	Male	65	IIIB	pT4N2	1/32	+++	+++	-	+
20	Male	66	IIIA	pT2bN2	4/8	+++	++	-	++
21	Male	67	IIB	pT2aN1	2/14	++	+++	-	+
22	Male	53	IIIA	pT2aN2	11/23	+++	+++	-	+
23	Male	59	IIIA	pT2aN2	16/31	++	+++	+	-
24	Male	45	IIIB	pT2aN3	8/11	+	+++	-	+
25	Male	60	IIIA	pT3N2	9/35	+++	++	-	-
26	Male	59	IIIA	pT2aN2	7/20	++	+++	+	-
27	Male	47	IIIA	pT2aN2	12/18	++	+++	-	+
28	Male	69	IIIA	pT2bN2	2/37	+	+++	+	++
29	Male	57	IIIA	pT3N1	1/11	+	+++	-	-
30	Male	60	IIIA	pT2aN2	5/11	++	+++	-	-
ERβ primary vs. LN *t* value			*p* value			Median score	Median score		
4.402			<0.001			11.10	14.57		

Addtionally, we further choosed 8-pair samples of NSCLC patients primary tumor tissues and metastatic lymph node tissue and analysised the protein expression between them. Similarly, the results showed that ERβ was overexpressed in NSCLC metastatic lymph node rather than it in primary tumor (Figure 1E). Thus, all these data suggest that ERβ expression was significantly higher in metastatic lymph node than in primary NSCLC tumor tissue.

### Expression of ERs and MMPs in NSCLC cell lines

**Figure 3:**
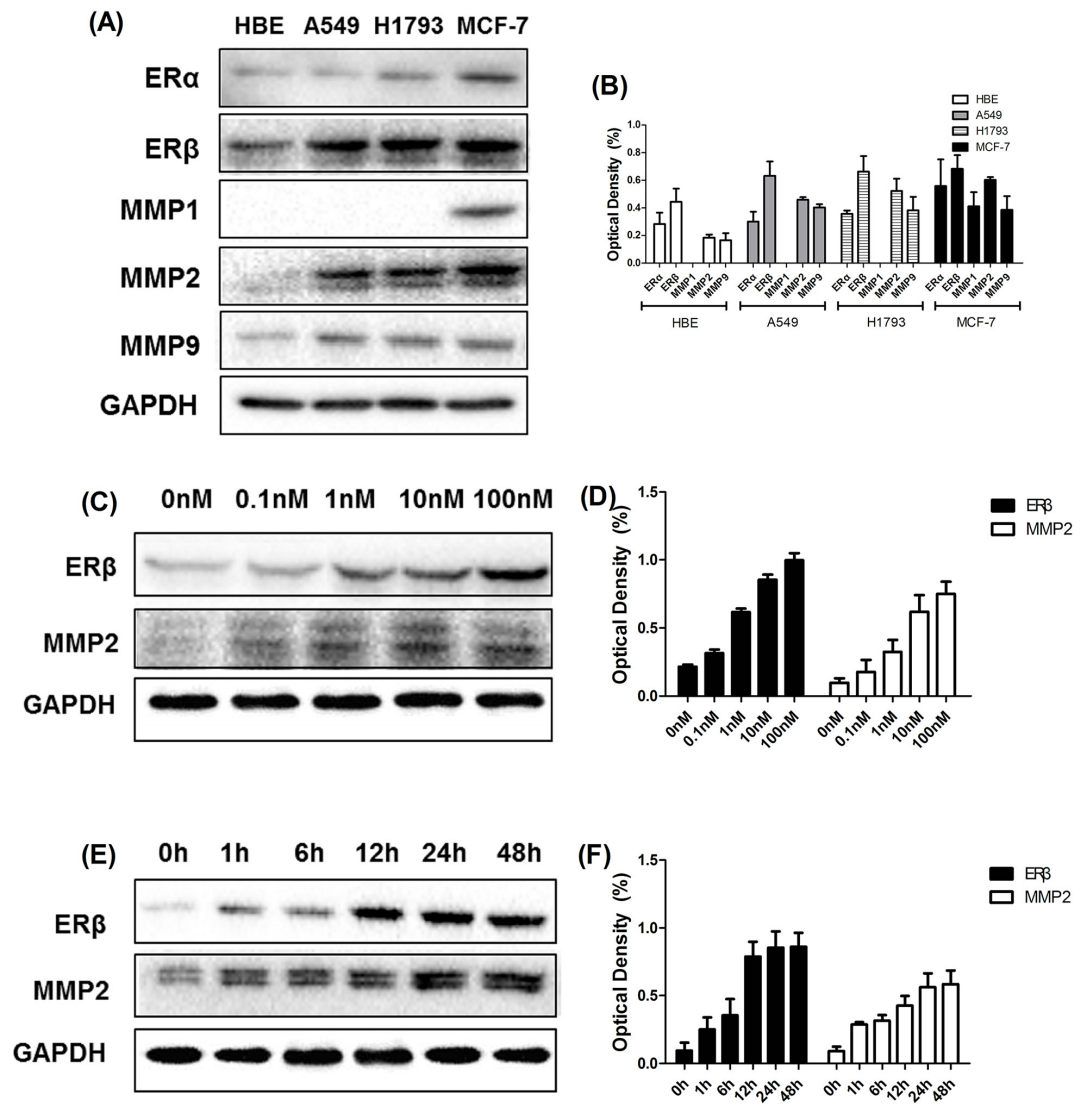
Western blotting analysis of ERβ and MMP-2 at different time points and different concentrations **(A-B)** Western blotting analysis of ERα, ERβ, MMP-1, MMP-2 and MMP-9 expression in cultured NSCLC cell lines (A549 and H1793) and breast cancer cell lines (MCF-7). **(C-D)** The synchronized cells were treated with E2 at different concentrations (0 nM, 0.1 nM, 1 nM, 10 nM and 100 nM) for 2 days. The protein expression of ERβ and MMP-2 was analyzed using Western blot. The data represent mean ± SEM from three different experiments. E2 stimulated both ERβ and MMP-2 response in A549 cell lines in a dose-dependent manner. **(E-F)** The synchronized cells were treated with E2 at different time points (0 h, 1 h, 6 h, 12 h, 24 h and 48 h) at concentrations of 10 nM. The protein expression of ERβ and MMP-2 was analyzed using Western blot. The data represent mean ± SEM from three different experiments. The data represent mean ± SEM from three different experiments. E2 stimulated both ERβ and MMP-2 response in A549 cell lines in a time-dependent manner. GAPDH was used as a control.

The expression of ERs and MMPs in normal bronchial epithelial cell line(HBE), NSCLC cell lines (A549 and H1793) and breast cancer (BC) cell lines (MCF-7) was evaluated using Western blot. As indicated in Figure 3A, ERβ was highly expressed in adenocarcinoma NSCLC cell lines A549 and H1793. In contrast, ERα was weakly expressed in NSCLC cell lines above. However, as positive controls, ERα and ERβ levels in BC cell lines MCF-7 were significantly higher compared with values reported in NSCLC cells. In addition, we also determined the expression of MMP-1, MMP-2 and MMP-9 in NSCLC cell lines, which were highly expressed in other studies of NSCLC [22, 23]. The expression level of ER and MMP was decreased in normal bronchial epithelial cell HBE than three kinds of tumor cells. Obviously, we found an elevated expression of MMP-2 and MMP-9 in MCF-7, A549 and H1793, while MMP-1 expression was only observed in breast cancer cell line.

### Effect of estrogen on the expression of ERβ and MMP-2 in NSCLC cell lines

Westren blots were performed in order to determine stimulation of ERβ and MMP-2 response by estrogen in NSCLC cell lines. We found that the expression of ERβ was significantly increased after E2 treatment in a dose- and time- dependent manner (Figure 3C-D). Interestingly, another similar dose- and time- dependent expression of MMP-2 was observed upon stimulation with E2 (Figure 3C-D). These results clearly indicated that E2 played an important role in increasing the expression of ERβ and MMP-2, which was highly correlated with E2 stimulation during cancer metastasis.

### Estrogen and ERβ agonist DPN increased ERβ and MMP-2 expression of NSCLC cells

**Figure 4:**
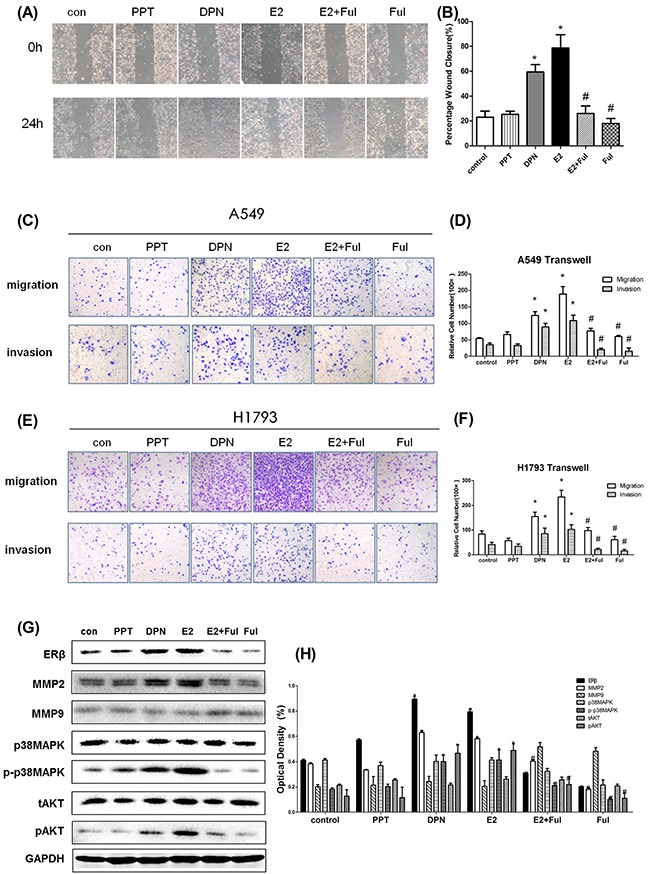
The aggressiveness of lung cancer cells under estrogen-related drug treatment **(A)** The migration in wound-healing assay of A549 cells after treatment with DMSO or E2 (10 nM), PPT (10 nM), DPN (10 nM), E2+Ful (1 μM) and Ful (1 μM) for 24 h (magnification 40×); **(B)** The effect on wound closure (percentage) in A549 cells (*p < 0.05 vs. control group, #p < 0.05 vs. E2 group); **(C)** Representative images of Transwell assays to assess A549 cell invasion and migration; **(D)** The effect of transwell migration assays in A549 cells after treating with DMSO or E2 (10 nM), PPT (10 nM), DPN (10 nM), E2+Ful (1μM) and Ful(1μM) for 24 h; The effect of transwell migration assays in H1793 cells after treatment with the same drugs for 72 h; **(E)** Representative images of Transwell assays for assessment of H1793 cell invasion and migration; **(F)** The effect of transwell migration assays in H1793 cells after treatment with DMSO or E2 (10 nM), PPT (10 nM), DPN (10 nM), E2+Ful (1 μM) and Ful (1 μM) for 24 h. The effect of transwell migration assays in H1793 cells after treatment with the same drugs for 72 h; **(G-H)** Western blot analysis of ERβ, MMP-2, MMP-9, p-p38MAPK, p38MAPK, pAKT and tAKT protein levels at 48 h in A549 cells, respectively. GAPDH was used as a control. All data are shown as the mean ± SD. Results represent three independent experiments. Student's t-test was carried out (*p < 0.05 vs. control group, #p < 0.05 vs. E2 group).

Since MMPs expression is critical for cancer metastasis, we investigated the effects of MMPs and correlated with ERβ levels. The effects of estrogen-like reagents and ER antagonists of ERβ and MMPs were investigated by treating A549 cells with DMSO (vehicle), E2, PPT, DPN, E2+Ful and Ful in different groups. The results showed that the expression of ERβ and MMP-2 was markedly increased in the E2 and DPN groups and was reduced after treatment with E2+Ful and Ful. However, no obvious changes were observed in PPT group compared with control. As illustrated in Figure 3A, MMP-1 was not expressed in NSCLC cell lines. E2 significantly increased the protein expression of ERβ and MMP-2, but not that of MMP-9. ERβ may control MMP-2 expression pre-translationally. Therefore, we also examined the key molecules downstream of p38MAPK and AKT pathways initially. As expected, the phosphorylation of p38MAPK and AKT induced by E2 was inhibited by Fulvestrant, while the total p38MAPK and AKT remained unchanged (Figure 4). Thus, these data strongly suggest that estrogen regulates MMP-2 by stimulating ERβ activity. ERα or MMP-9 may not act as key regulatory factors in cancer progression.

### ERβ upregulated by estrogen and ERβ agonist DPN enhances aggressiveness of lung cancer cells

After upregulation of MMP-2 by estrogen, we investigated whether ERβ overexpression enhanced the migration and invasion of NSCLC cells. NSCLC cell migration and invasion were evaluated by wound-healing assay, transwell migration, and invasion assay. Wound-healing assay revealed that cells treated with E2 and DPN significantly increased the rate of lateral migration into a wound introduced in a confluent monolayer of cells compared with control groups and inhibitor groups (Figure 4A-B, p < 0.05). Consistently, transwell migration assay also showed that cells after E2 and DPN treatment promoted cellular transmigration compared with controls (p < 0.05). Transwell invasion assay further demonstrated that E2 and DPN treatment significantly increased the number of cells that penetrated through the Matrigel-coated membrane (p < 0.05). Meanwhile, the PPT group, treated with a specific ERα agonist, remained unchanged In migration and invasion compared with controls. In addition, we found that the combined treatment with E2 and Ful, and Ful alone resulted in a significant decrease in the number of invading cells compared with the effects of control group (Figure 4C-F, p < 0.05). Therefore, these results clearly demonstrated that NSCLC cells are more aggressive in migration and invasion during ERβ and MMP-2 overexpression induced by estrogen.

### ERβ regulate NSCLC cell invasion and migration, and interaction with MMP-2

**Figure 5:**
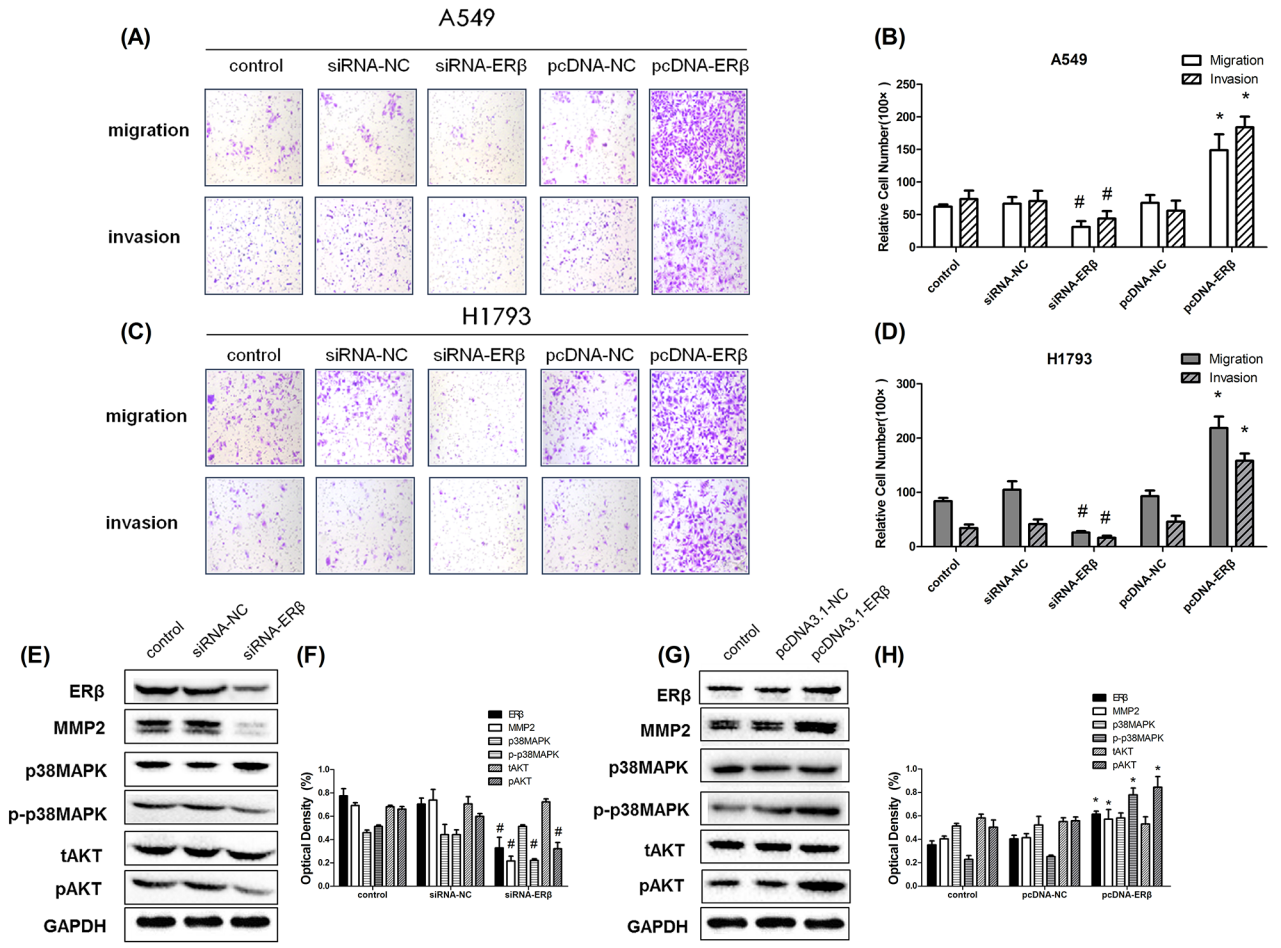
The aggressiveness of lung cancer cells under RNAi intervention **(A-B)** Representative images of Transwell assays to assess A549 cell invasion and migration. The effect of transwell migration and invasion assays with decreased protein level of ERβ in siRNA-ERβ cells compared with siRNA-NC, increased protein level of ERβ in pcDNA-ERβ cells compared with pcDNA-NC and control group; **(C-D)** Representative images of Transwell assays to assess H1793 cell invasion and migration: The effect of transwell migration and invasion assays with decreased protein level of ERβ in siRNA-ERβ cells compared with siRNA-NC, increased protein level of ERβ in pcDNA-ERβ cells compared with pcDNA-NC and control group; **(E)** Decreased protein level of ERβ in siRNA-ERβ A549 cells compared with siRNA-NC A549 cells by Western blot and **(F)** the detection of optical density; **(G)** Increased protein level of ERβ in pcDNA-ERβ A549 cells compared with pcDNA-NC A549 cells by Western blot and **(H)** the detection of optical density. GAPDH was used as a control. All data are shown as the mean ± SD. Results were representative of three independent experiments. Student's t-test was carried out (#p < 0.05 vs. siRNA-NC group*p < 0.05 vs. pcDNA-NC group).

To explore the mechanisms of ERβ regulation of NSCLC cell migration and invasion, we assessed the aggressiveness of NSCLC cell by overexpressing and repressing ERβ. The effect of siRNA transfection and plasmid transfection on the ERβ expression was confirmed by Western blot. Cell migration and Matrigel® coated Transwell® invasion assays clearly revealed that ERβ silencing in parental A549 cells strongly reduced cell migration and invasion (Figure 5A-B). Sequentially, we upregulated ERβ expression using pcDNA3.1-ERβ vectors in A549 cells. As expected, it significantly promoted cell migration and Matrigel®-coated Transwell® invasion was observed in ERβ-overexpressing A549 cells compared with the pcDNA3.1-control group (p < 0.01, respectively (Figure 5A-B). Similar result was obtained in the parallel experiment using another NSCLC cell line H1793 (Figure 5C-D). Our data indicate that ERβ expression is associated with the aggressive NSCLC cell migration and invasion. Concurrently, to investigate the role of ERβ in the stabilization of MMP-2 in NSCLC cells, we examined whether the expression of MMP-2 was altered during ERβ regulation. The results show that overexpression of ERβ in NSCLC cells increased the expression of MMP-2 and ERβ depletion resulted in down-regulation of MMP-2 (Figure 5E-H). Thus, these data strongly suggest that ERβ regulates MMP-2 activation in NSCLC metastasis.

### p38MAPK/AKT signaling pathways enhanced the expression of MMP-2 mediated by ERβ

**Figure 6:**
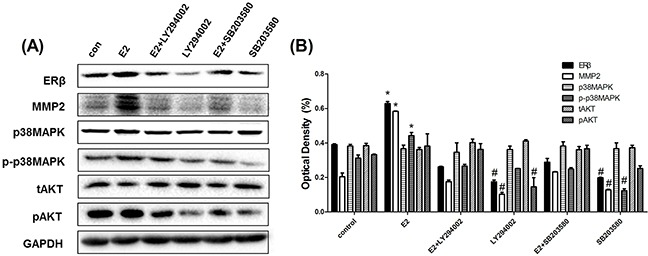
pAKT inhibitor(LY294002) and p-p38MAPK inhibitor(SB203580) significantly suppressed ERβ and MMP-2 expression in NSCLC cell lines **(A)** Western blot analysis of protein expression of ERβ, MMP-2, p-p38MAPK, p38MAPK, pAKT and tAKT in NSCLC cell line A549 when treating with DMSO or E2 (10 nM), E2+LY294002 (10 nM), LY294002 (10 nM), E2+SB203580 (10 nM) and SB203590 (10 nM) for 48 h. **(B)** the detection of optical density. GAPDH was used as a control. All data are shown as the mean ± SD. Results represent three independent experiments. Student's t-test was carried out (*p < 0.05 vs. control group, #p < 0.05 vs. E2 group).

Considering the fact that ER signaling in cancer requires activation of the downstream MAPK and Akt pathways, we further determined whether the phosphorylation of p38MAPK and AKT was activated by ERβ regulation. As mentioned before, 10 nM E2 induced the phosphorylation of p38MAPK and AKT, which was inhibited by Fulvestrant. When ERβ expression in parental A549 cells was silenced by siRNA transfection, the protein level of phosphorylated p38MAPK and AKT decreased. Conversely, increased phosphorylation levels of p38MAPK and AKT were observed during ERβ overexpression in NSCLC cells. However, the total expression of p38MAPK and AKT remained unchanged during ERβ over-expression or inhibition (Figure 5E-H). Furthermore, we choosed pAKT inhibitor(LY294002) and p-p38MAPK inhibitor (SB203580) to treat NSCLC cell line and finally found that ERβ/MMP-2 was significantly abrogated when pAKT and p-p38MAPK was suppressed. Besides, in this study, pAKT was shown to make more principal effect than p-p38MAPK (Figure 6A-B). These data suggest that ERβ may regulate MMP-2 activity via the p38MAPK and AKT signaling pathway in lung cancer cells.

### Estrogen induces metastasis of A549 human NSCLC cells *in vivo* while fulvestrant suppresses the metastatic effect

**Figure 7:**
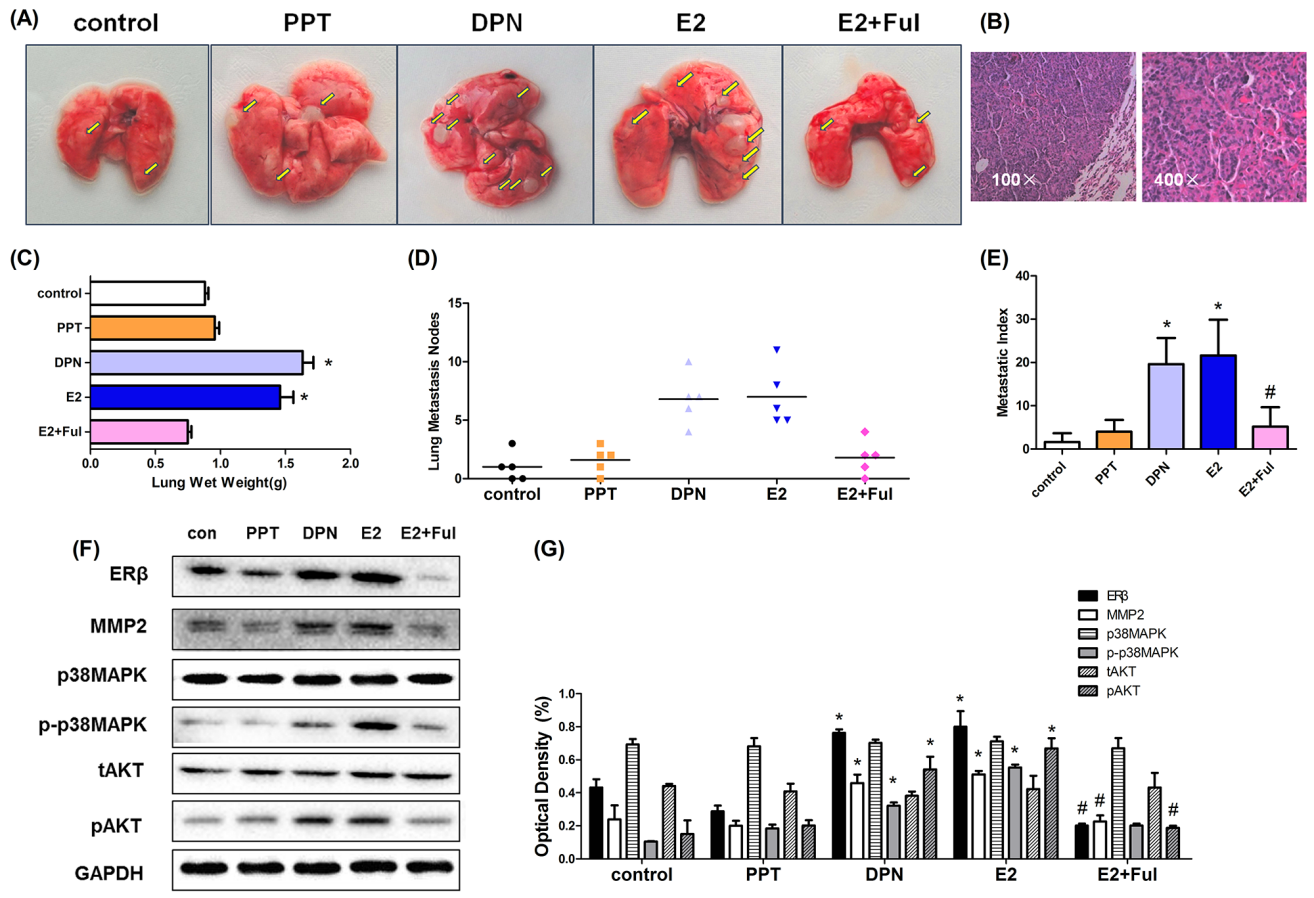
Estrogen and DPN promote lung metastasis of A549 human non-small cell lung cancer cells *in vivo* while Fulvestrant suppressed the metstasis **(A)** After bilateral ovariectomy A549 cells (5 × 10^6^/100 μL) suspended in PBS were injected into 4-week-old female BALB/c nude mice via the tail vein, mice were randomly divided into five groups (N = 5/group): E2(0.09 mg/kg), PPT(1.05 mg/kg), DPN(1.89 mg/kg), E2+ Ful(1.46 mg/kg) and blank control. The lungs were removed after 4 weeks subcutaneously under drug treatment. Gross appearance of metastatic lung tumor nodes in different groups is indicated by bright yellow arrows. **(B)** Representative pathological image of metastatic A549 tumor at magnification 100ificatione pathological image of groups (N = 5/group): The number of metastatic nodules in the lungs (lung nodules number in every mouse of each group), metastatic index in different groups mentioned in ‘‘Materials and methods’’ section for the experimental. **(C-E)** The mean lung wet weight of each group, The number of metastatic nodules in the lungs (lung nodules number in every mouse of each group), metastatic index in different groups mentioned in ‘‘Materials and methods’’ section for the experimental. **(F)** Protein expression of ERβ, MMP-2, MMP-9, p-p38MAPK, p38MAPK, pAKT and tAKT in murine lung metastatic nodes was analyzed using Western blot, and **(G)** the detection of optical density. All data are expressed as the mean ± SD. Student's t-test was carried out (*p < 0.05 vs. control group, #p < 0.05 vs. E2 group).

In order to confirm whether estrogen promoted metastasis of A549 cells in an experimental lung metastatic mouse model, estrogen, PPT, DPN and Ful were injected subcutaneously into BALB/C nude ovariectomized mice bearing A549 cancer cells twice weekly. After 45 days of treatment, mice from each group (n = 5) were scarified and the number of metastatic nodules in the lungs was examined at the indicated time points. The numbers of lung metastatic lesions in E2 and DPN groups were significantly higher than in the control group. However, no obvious differences were seen in PPT, E2+Ful, and control groups (Figure 7A-B). Increased lung weight and metastatic index, reflecting the volume and number of metastatic nodes, were observed in E2 and DPN groups compared with control (Figure 7C-E).

Western blot was used to demonstrate the expression of ERβ and MMP-2 in mouse tumor tissue. The expression of ERβ and MMP-2 in the E2 and DPN groups was significantly higher than in the control group and decreased in E2+Ful group. Compared with the control, we also found increased phosphorylated p38MAPK and AKT levels following stimulation by E2 and DPN. However, the protein expression was unchanged by PPT stimulation (Figure 7F-G). Thus, estrogen and ERβ agonist DPN promoted lung metastasis of A549 cells *in vivo* by activating ERβ via p38MAPK and AKT signaling pathway.

## DISCUSSION

Estrogen is synthesized locally in lung tumors, and aromatase is highly expressed in lung tumor tissue [25, 26]. Preclinical data demonstrated that estrogen induced lung cancer [27]. Several studies also demonstrated that ERβ is the dominant ER in the development of human NSCLC [12, 28, 29]. Moreover, in the cytosol estrogen induces rapid signaling via Src kinase, MAPK and AKT pathways [18, 19]. The present study revealed that estrogen induces cell proliferation of NSCLC cells *in vitro* [13], induces tumor formation in human tumor xenografts [12] and in urethane-induced NSCLC animal models [30], raising the question whether estrogen-induced ERβ even can promote lung cancer metastasis and the underlying mechanisms and signal transduction pathways activated.

In a previous cohort study of 183 American NSCLC patients, the cytoplasmic ERβwas found to be an independent poor prognostic factor for lung cancer [28]. Our study population including 222 Chinese NSCLC patients revealed that the high expression of ERβ was significantly correlated with poorer tumor differentiation and distant metastasis, which predict poor prognosis in the malignant tumor. In our study, due to the significant loss ratio of follow-up (overall survival follow-up loss ratio = 34.96%; disease-free survival follow-up loss ratio = 46.38%), we failed to show the results of prognostic analysis of ERβ in this study. The prognostic data of this population will be further investigated. However, when we grouped different score of ERβ using IHC staining, we found that the proportion of patients suffering from lymph node metastasis was higher in the ERβ-positive and intensely staining group. Thus, the results provide evidence suggesting that elevated ERβ expression correlated with tumor metastasis in NSCLC.

Data reveal that the expression of ERβ was significantly higher in metastatic lymph node than in the primary NSCLC tumor tissue, which further suggests that ERβ activation may enhance metastatic aggressiveness of lung cancer cells. Due to the relative scarcity of metastatic lymph nodes available for labor experiment, it is the first time that the differential expression of ERβ in primary tumor and metastatic lymph nodes derived from the same NSCLC patient was observed. Additionally, two studies focus on *EGFR* mutation in NSCLC metastastic lymph node, in which differential gene expression in primary lung tumor tissues and matched metastatic lymph nodes were reported [31, 32]. Remarkably, two major models of tumor metastasis including parallel progression and linear progression, provide extremely different views on tumor metastasis. The “parallel progression model” predicts very early dissemination of cancer cells to distant organs with highly diverse genetic profiles of the primary and metastatic tumors. The “linear progression model” suggests metastasis as a relatively late event of tumor progression [20, 31]. Based on our results, ERβ are strongly expressed in metastatic lymph node, we assumed that NSCLC cells, which are ERβ-overexpressed may have great potential to metastasize to lymph nodes or distance organs and eventually form macrometastases. This hypothesis might explain the phenomenon observed in our experiment, which supports the “parallel progression model”. However, two cases of relatively lower expression of ERβ occurred in metastatic lymph node but were expressed higher in the primary tumors suggesting that the molecular status may be altered after metastasis. Taken together, our findings suggest that the differential ERβ activation including low expression in primary lung tumor tissues but high expression in matched metastatic lymph node, was one of the possible factors inducing NSCLC lymph node metastasis.

*In vitro*, after confirming that E2 promoted aggresiven migration of NSCLC cells in wound-healing and transwell assays, we investigated whether ERα or ERβ played a leading role in tumor metastasis by different estrogen related drugs. Results showed that only DPN significantly enhanced the expression of ERβ, cell migration and invasion but not PPT. Moreover, when treated with Fulvestrant or silenced with siRNA-ERβ, we observed a significant decrease in aggressive property of NSCLC cells and in tumor formation of murin lung cancer metastasis model. Consistent with our results, prior studies demonstrated that estrogens promote lung cancer development and induce activation of ERβ [12, 14, 15, 28]. *In vivo*, the tumor formation rate of A549 cells induced by E2 and DPN was greater and more extensive compared with the control group. Treatment with ER-antagonist Fulvestrant resulted in a significantly decreased lung metastasis. Tang, et al [15] and Liu,et al [24] developed a urethane-induced murine adenocarcinoma model to demonstrate the role of estrogen *in vivo*, consistent with our study. Therefore, these results imply that upregulated ERβ promoted NSCLC cell proliferation and metastasis.

Metastasis is a complex multistep process that includes degradation of the basement membrane and the extracellular matrix [33, 34]. Epithelial cancer metastasis through the basement membrane and stroma is frequently mediated via matrix-metalloproteinase (MMP) [35]. MMP family of proteinases, particularly MMP-1, MMP-2 and MMP-9 expressed in lung cancer [22, 23], play an important role in malignant tumor metastasis [34]. Therefore, we stained MMP-1, MMP-2 and MMP-9 in 222 NSCLC specimens in the TMA study. MMP-1, MMP-2 and MMP-9 were all increased in tumor samples compared with matched, corresponding normal tissues. However, it significant correlation was found only between ERβ and MMP-2 in tumor tissues. Based on these results, we first combined the key molecular mechanisms underlying estrogen-induced NSCLC metastasis, with MMP-2. Gonzalez-Arriaga, P.et al reported increased expression of MMP-2 in NSCLC tumor tissue, highlighting the role of MMP-2 in tumor progression [23]. We observed a dose- and time-dependent expression of ERβ closely related to MMP-2 *in vitro*. Then, we investigated the effect of MMP-2 overexpression or silencing on ERβ expression and aggressive lung cancer. The results suggest that ERβ overexpression increased MMP-2 expression level, while ERβ silencing had the opposite effect. The results of metastatic lung cancer murine model further revealed that ERβ and MMP-2 were similarly overexpressed in E2 and DPN groups. These results suggested that estrogen promoted lung cancer metastasis via ERβ-induced upregulation of MMP-2.

Extranuclear signaling has been linked to rapid responses to E2 via stimulation of mitogen-activated protein kinase (MAPK), especially p38MAPK, protein kinase B (AKT), phosphatidylinositol-3-kinase (PI3K) in the cytosol [18, 19]. Mechanistically, considering the fact that a key biological function of MAPK/AKT is regulation of the expression of MMPs [36–39], we found that MMP-2 activation induced by ERβ were significantly associated with p38MAPK and AKT phosphorylation *in vitro* and *in vivo*. Taken together, these results demonstrated that estrogen regulates MMP-2 expression via ERβ and may promote metastasis through the p38MAPK and PI3K/AKT signaling pathway.

In conclusion, we revealed evidently that estrogen promotes NSCLC tumor metastasis through ERβ by increasing MMP-2, and p38MAPK and AKT activities also should be considered as molecular parameters in this progression. After proving estrogen plays an important roll in NSCLC cell proliferation in previous works, now we further reveal the significant effects of estrogen in NSCLC metastasis. Thus, targeting ERβ should be evaluated as an appealing strategy for advanced lung cancer patient therapy, particulally for whom suffering metastasis.

## MATERIALS AND METHODS

### Patient characteristics and metastatic lymph node specimens: tissue microarray (TMA) preparation

Between October 2008 and February 2014, 222 paraffin-embedded, formalin-fixed, surgical specimens of pathologically diagnosed primary non-small cell lung cancer tissues were obtained from the Department of Thoracic Surgery, Affiliated Tongji Hospital of Huazhong University of Science and Technology Tongji Medical College (Wuhan, China). TMA was prepared by Zuocheng Biotech (Shanghai, China). None of the patients underwent prior chemotherapy or radiotherapy before surgery. Patients’ demographics including gender, age, smoking history, pathological diagnosis, pTNM stage and tumor differentation grade were obtained from the Tongji hospital records. Baseline characteristics of the patients are shown in Table 1. Table 2 reveals the association between ERβ with MMP-1, MMP-2 and MMP-9 expression in the 222 NSCLC tissue microarray. In these patients, metastatic lymph nodes were obtained from 30 cases involving paired primary tumors via surgical resection of primary tumor with lymph node dissection. Cases of lymphadenitis and primary malignancies of lymph node were excluded. Details of the 30 IIA-IIIB NSCLC cases with lymph node metastasis are included in this study (Table 3). Each metastatic lymph node represented the primary tumor tissue referred to above. Palliative care or surgical biopsy following informed consent was administered to 38 cases of patients with inoperable Stage IIIb–IV primary NSCLC.

To evaluate the contribution of ERβ activity and its association with MMPs and clinical pathology of NSCLC patients, adjacent nontumor tissue of patients undergoing surgical intervention was used as normal control. None of these patients showed abnormal hepatic or renal function, or endocrine abnormalities. They also did not receive prior steroid treatment, chemotherapy or radiotherapy within the past months. All the carcinomas were classified according to the p-TNM staging system (UICC, 7th edition, 2009). All the cases provided histopathology specimens for assessment and further management. Informed consent was obtained before surgery. All the slides were coded and molecular studies were investigated without knowledge of the patient's identity or clinical status. This study was approved by the Research Ethics Committee, Tongji Medical College, Huazhong University of Science and Technology (The IRB ID number is 20141101).

### Immunohistochemical(IHC) staining for ERβ/MMPs expression in NSCLC tissues

Immunohistochemical staining of ERβ/MMPs was carried out using the avidin–biotin peroxidase method. We boiled the tissue sections for 5 min in a high-pressure cooker before immunohistochemical staining to perform antigen retrieval using the protocol mentioned before [14, 29]. All the sections were de-paraffinized in xylene, rehydrated in alcohol, incubated in hydrogen peroxide, blocked in 10% goat serum (Bei Jing Zhong Shan-Golden Bridge Technology Co., Ltd, China) and incubated with anti polyclonal antibody. Rabbit anti-human ERβ(1:200, Proteintech, CA, USA), mouse anti-human MMP-1(1:200, Santa Cruz Biotechnology, CA, USA), mouse anti-human MMP-2 (1:200, Santa Cruz Biotechnology, CA, USA), and rabbit anti-human MMP-9(1:500,Proteintech, CA, USA) antibodies were used for staining. After overnight incubation at 4°C, the slides were incubated with general immunohistochemical secondary antibodies (DAKO) for 30 min at 37°C and visualized with DAB. Staining scores were evaluated pathologically in a double-blinded manner under a light microscope. The immunoreactivity scores of the cancer tissue samples were determined based on the staining intensity and area of positive staining according to the method described in Tang et al [15]. Positive cells were scored as follows: (1) ≤ 20 % positive cells; (2) 20–50 % positive cells; (3) 50–75 % positive cells; and (4) > 75 % positive cells. Staining intensity was evaluated as follows: (1) negative; (2) weakly positive; (3) moderately positive; and (4) strongly positive. A score of 1–16 was obtained by multiplying the staining intensity and proportion of positive cells: (-), ≤ 4; (+), > 4 and ≤ 8; (++), > 8 and ≤ 12; and (+++), > 12 and ≤ 16. A total score > 4 was defined as positive expression, and a score ≤ 4 was defined as negative.

### Cell lines, plasmids, RNAi and transfection

A549 and H1793 cells were the two NSCLC cell lines used in this study. They were purchased from the American Type Culture Collection and grown for 2 weeks (four passages) before freezing aliquots for subsequent analyses using the same batch of personally passaged cells. A549 cells were cultured in Roosevelt Park Memorial Institute Medium (RPMI 1640) supplemented with 10% fetal bovine serum (FBS). H1793 cells were cultured in DMEM:F12 medium supplemented with 10 % FBS, 0.005 mg/mL insulin, 0.01 mg/mL transferrin,30 nM sodium selenite, 10 nM hydrocortisone, and extra 2 mM L-glutamine. All the cells were maintained at 37°C in a humidified incubator with 95% air and 5% CO_2_.

The ERβ-expressing plasmid and its empty plasmid were obtained from Origene Technologies Inc. (Rockville, USA). Small interfering RNAs targeting ERa (ERa-siRNA Stealth siRNAs HSS103378, Life Technologies, Carlsbad, CA) and control siRNAs (ctrl-siRNA, Life Technologies, Carlsbad, CA) were constructed using a gene silencing vector. Lipofectamine 3000 reagent were used during transfection following the manufacturer's instructions. ERβ protein expression was analyzed using Western blot following transfection with plasmids or siRNAs.

### Drug treatment

The parental NSCLC cells or mouse metastatic model were treated with17β-estradiol, known as E2 (Sigma-Aldrich, St. Louis, MO, USA), 4,40,400-(4-propyl-[1H]-pyrazole-l,3,5-triyl) trisphenol (PPT, an ERα agonist, Sigma-Aldrich), 2,3-bis(4-hydroxyphenyl) propionitrile (DPN, an ERβ agonist, Sigma-Aldrich), fulvestrant (Ful, an ER antagonist , Cayman Chemical) either alone or as a combination. The dosages of these drugs used *in vitro* and vivo are comparable as previously described [14, 24, 40, 41]. Each group of cells was treated for 48 h and harvested for further analysis. Cell culture experiments were performed using reagents formulated in 100 % DMSO.

### Western blot

Western blots were performed as described pre-viously [24]. Briefly, the total protein extract of each tissue sample or cell line was dissolved in a lysis buffer (25 mM Tris–HCl pH 7.4,1 % Triton X-100, 150 mM NaCl, 5 % EDTA, 10 mM NaF, 1 mM phenylmethylsulfonyl fluoride, and 10 mg of aprotinin and leupeptin), separated on 10% SDS-PAGE gels, transferred to PVDF membranes (Millipore, USA) and quantitated using a BCA protein assay kit. Equal amounts of protein (60 μg) were analyzed by immunoblotting. The antibodies used during Western blots included rabbit anti-human ERα from Proteintech, rabbit anti-human ERβ (1:500) from Proteintech, mouse anti-human MMP-1(1:400) from Santa Cruz, mouse anti-human MMP-2(1:400) from Santa Cruz, rabbit anti-human MMP-9 (1:500) from Proteintech, rabbit anti-human p38MAPK(1:500)/p-p38MAPK(1:500) and rabbit anti-human tAkt (1:500)/pAkt(1:500) from Bioworld Technology. Rabbit anti-human GAPDH (1:5000) from Santa Cruz was used as an internal control for protein loading and analysis. The protein bands were visualized using enhanced chemiluminescence (ECL) kit (Pierce, USA).

### Wound-healing assay

NSCLC cells (5 × 10^5^/mL) were seeded into six-well plates and cultured under standard conditions. A wound was created by scraping the cell monolayer with a 1000 μL pipette tip when the cells reached confluence. Cell migration was determined by measuring the movement of cells into the scraped area. Representative images (40×) of wound closure were captured at 0 h and 24 h using an inverted light microscope.

### Invasion and migration assay

The invasive ability of cells was analyzed using Matrigel™ (BD) and Transwell® (Corning) assays. The upper chamber of Transwell® was filled with 50 μL of a mixture of Matrigel™ and serum-free RPMI 1640 or DMEM:F12 medium (at a ratio of 1:3), which was then transferred to a 37°C incubator until the water evaporated. The Matrigel™ was hydrated using basic medium for 30 min before the addition of cells. A549 and H1793 cells were treated or transfected for 48 h and resuspended at a density of 1×10^5^/mL in serum-free RPMI-1640 or DMEM:F12 medium. Approximately 2×10^4^ cells/well were seeded in the upper chamber, and the lower well was filed with 500 μL RPMI 1640 or DMEM:F12 supplemented with 10% FBS. After incubation for 48 h at 37°C with 5 % CO_2_, any cells remaining in the upper chamber were removed. The cells in the lower chamber were fixed with 4% paraformaldehyde and stained with crystal violet for 10 min at room temperature. Similar experimental procedure was used for migration assay, with the exception that the filters in upper well were not pre-coated with Matrigel. The invasion and migration were evaluated based on the number of cells invading the Traswell® membrane, which was counted using an Olympus microscope (Olympus, Tokyo, Japan) at 100× magnification. Four fields were randomly selected for analysis.

### Experimental model of murine lung metastasis

An experimental model of A549 lung metastasis was used to investigate the effects of estrogen on the development of metastases *in vivo*. Following bilateral ovariectomy to eliminate the effect of endogenous estrogen, A549 cells (5 × 10^6^/100 μL) suspended in PBS were injected into 4-week-old female BALB/c nude mice via the tail vein. Seven days after tumor injection, mice were randomly divided into five groups (N = 5/group): E2 (0.09 mg/kg), PPT (1.05 mg/kg), DPN (1.89 mg/kg), E2+ Ful (1.46 mg/kg), and blank control. Drugs were subcutaneously injected twice weekly in a volume of 100 μL per mouse, respectively. After 4 weeks of treatment, the lungs were removed and the number of metastatic nodules was counted under a dissecting microscope.

Lungs were fixed, embedded, and sectioned. A portion of the lung metastatic nodules was fixed in 10 % formalin, embedded in paraffin, and sectioned for IHC. Other lesions were stored in liquid nitrogen and later at −80°C for further use (Western blot). The total number of lung nodules, lung wet weight, and lung cancer metastatic index were statistically analyzed. The “Metastatic Index”, which was developed by D. Deodhar, et al [42],was used to approximately assess the total metastatic tumor burden in the lungs. Each metastasis measuring less than 0.5 mm in diameter was graded as I, between 0.5 and 1 mm as Grade II, between 1.0 and 2 mm as Grade III, and >2 mm as Grade IV. All the grade scores of total lung lobes were added to determine the metastatic index for a given animal. The mean index was calculated for a given control or experimental group of animals.

Female Balb/c nude mice (Animal Purchase No.43004700006938) were obtained from the Experimental Animal Center of Hubei Province (Animal Study Permit No. SCXK 2013-0004) and maintained in an environment with a standardized barrier system (System Barrier Environment No. 00127070) in the Experimental Animal Center of Tongji Hospital of Huazhong University of Science and Technology. The above parameters were determined by two qualified technicians in a double-blinded manner, and the average of the two scores was obtained.

### Statistical analysis

Data were expressed as the mean ± SEM. Statistical significance was established, using the SPSS 19.0 statistical software package (SPSS Inc., Chicago, IL, USA). Associations between ERβ expression and clinicopathological determinants were evaluated using the χ2 test and Fisher's exact test, as appropriate. Correlation between ERβ and MMPs expression in NSCLC was analyzed using Spearman's rank correlation. Differences in the expression of ERβ and MMPs between primary tumor and its metastatic lymph node were examined using the t-test of Paired Samples. The other data *in vivo* and *in vitro* were analyzed using Student's t-test to evaluate significant differences. A p-value of < 0.05 was considered statistically significant.
